# Long-term outcomes of surgical resection for T1b gallbladder cancer: an institutional evaluation

**DOI:** 10.1186/s12885-019-6507-2

**Published:** 2020-01-06

**Authors:** Kizuki Yuza, Jun Sakata, Pankaj Prasoon, Yuki Hirose, Taku Ohashi, Koji Toge, Kohei Miura, Masayuki Nagahashi, Takashi Kobayashi, Toshifumi Wakai

**Affiliations:** Division of Digestive and General Surgery, Niigata University Graduate School of Medical and Dental Sciences, 1-757 Asahimachi-dori, Niigata City, 951-8510 Japan

**Keywords:** Gallbladder neoplasms, Surgery, Prognosis, Treatment outcome

## Abstract

**Background:**

There is no comprehensive agreement concerning the overall performance of radical resection for T1b gallbladder cancer (GBC). This research focused on addressing whether T1b GBC may spread loco-regionally and whether radical resection is necessary.

**Methods:**

A retrospective analysis was conducted of 1032 patients with GBC who underwent surgical resection at our centre and its affiliated institutions between January 1982 and December 2018. A total of 47 patients with T1b GBC, 29 (62%) of whom underwent simple cholecystectomy and 18 (38%) of whom underwent radical resection with regional lymph node dissection, were enrolled in the study.

**Results:**

GBC was diagnosed pre-operatively in 16 patients (34%), whereas 31 patients (66%) had incidental GBC. There was no blood venous or perineural invasion in any patient on histology evaluation, except for lymphatic vessel invasion in a single patient. There were no metastases in any analysed lymph nodes. The open surgical approach was more prevalent among the 18 patients who underwent radical resection (open in all 18 patients) than among the 29 patients who underwent simple cholecystectomy (open in 21; laparoscopic in 8) (*P* = 0.017). The cumulative 10- and 20-year overall survival rates were 65 and 25%, respectively. The outcome following simple cholecystectomy (10-year overall survival rate of 66%) was akin to that following radical resection (64%, *P* = 0.618). The cumulative 10- and 20-year disease-specific survival rates were 93 and 93%, respectively. The outcome following simple cholecystectomy (10-year disease-specific survival rate of 100%) was equivalent to that following radical resection (that of 86%, *P* = 0.151). While age (> 70 years, hazard ratio 5.285, *P* = 0.003) and gender (female, hazard ratio 0.272, *P* = 0.007) had a strong effect on patient overall survival, surgical procedure (simple cholecystectomy vs. radical resection) and surgical approach (open vs. laparoscopic) did not.

**Conclusions:**

Most T1b GBCs represent local disease. As pre-operative diagnosis, including tumour penetration of T1b GBC, is difficult, the decision of radical resection is justified. Additional radical resection is not required following simple cholecystectomy provided that the penetration depth is restricted towards the muscular layer and that surgical margins are uninvolved.

## Background

Recognition of early-stage gallbladder cancer (GBC) remains difficult. Approximately 50% of GBCs are discovered by pathological examination following simple cholecystectomy for assumed benign gallbladder disease [1, 2]. Recently, the likelihood of early-stage GBC, which is restricted to tumour infiltrating the lamina propria (T1a) or muscular layer (T1b), has grown as a result of the extensive use of laparoscopic cholecystectomy for benign gallbladder disease, which will also increase in the foreseeable future [3]. Early-stage GBC has a comparatively good prognosis and can be treated by simple cholecystectomy or extended cholecystectomy. For T1a GBC, simple cholecystectomy is mostly adequate without any need for additional procedures, providing the resected margin is just not concerned [4].

Nevertheless, the type of operative procedure for T1b GBC continues to be contentious. The explanation for this difference in operative approach might be that the likelihood of GBC is comparatively minimal, which makes it challenging to obtain adequate cases to provide treatment guidelines for each stage, and the prognostic outcomes of GBC may differ on account of variations in regional occurrence [5]. Furthermore, the gallbladder wall is very thin, and it is quite difficult to histologically evaluate, and dysplasia is presented as inflammation, which makes the proper diagnosis of cancer and T stage complicated, leading to variations in pathological reviews from centre to centre [6, 7].

Controversy persists over whether T1b GBC may have spread locally, regionally, or systemically at presentation. This uncertainty leads some surgeons to perform extended cholecystectomy in preference to simple cholecystectomy [8]. The National Comprehensive Cancer Network (NCCN) guidelines indorse radical resection with portal lymph node dissection for T1b GBC [9], whereas the Japanese guidelines recommend simple cholecystectomy provided that the depth of invasion is histologically restricted to the muscular layer [10]. The choice to carry out additional resection is often based upon pathological results through the preliminary operatively resected specimen, in which the operative margin status, nodal status and/or lympho-vascular invasion essentially indicates the most potential residual disease.

Experts have formerly documented that the outcome following simple cholecystectomy was fairly exceptional, and therefore, added radical resection was not required in 25 patients with T1b GBC [11]. On the other hand, the small number of patients and shorter follow-up time precluded a conclusive final result. The purposes of this study were to address whether T1b GBC may spread loco-regionally and to appraise the long-term outcomes of simple cholecystectomy and radical resection with regional lymph node dissection for T1b GBC adhering to our institutional pathological results to determine the preferred treatment.

## Methods

### Study subjects

A total of 1032 patients who underwent surgical removal of GBC in Niigata University Medical and Dental Hospital and its affiliated institutions between January 1982 and December 2018 were reviewed retrospectively. A total of 290 (27%) had pT1a tumours, and 49 (4.6%) had pT1b tumours. Two patients were omitted from the present study because of concomitant advanced intrahepatic cholangiocarcinoma (*N* = 1) and pancreatic cancer (*N* = 1). The remaining 47 patients with pT1b disease were included in this retrospective study. There were 31 women and 16 men with ages ranging from 38 to 94 (median, 74) years. This study was permitted by the Institutional Review Board of the Niigata University Graduate School of Medical and Dental Sciences, Niigata, Japan (2018–0137). All patients included in this study provided written consent for treatment. Patients were not needed to provide well informed consent to this research because the evaluation applied unidentified clinical information.

### Diagnosis and surgical treatment

The choice of procedure relied on the timing of diagnosis. Among the 47 patients, GBC was diagnosed prior to operation by ultrasonography in 16 patients (34%), fungating mass in 9 patients and a smooth raised mass in 7 patients, conferring to Tsuchiya’s classification [12]. Ultrasonographic evaluation of the depth of invasion was not feasible for most of the 16 patients prior to the operation. Of the 16 patients, 14 underwent radical resection with regional lymphadenectomy, and 2 patients underwent simple cholecystectomy alone because of poor general condition and associated comorbidities.

The remaining 31 patients (66%) had incidental GBCs, which were discovered by operative findings, operative ultrasonography, or both (*N* = 8) or by post-operative pathological examination (*N* = 23) after simple cholecystectomy for presumed benign disease of the gallbladder. The pre-operative diagnosis of 31 patients with incidental GBC included polypoid lesions of the gallbladder in 9, adenomyomatosis in 1, and gallstone disease or cholecystitis in 21. Of the 8 patients who had an intra-operative diagnosis of GBC, 6 underwent simple cholecystectomy alone, and 2 underwent radical resection with regional lymphadenectomy as a consequence of conversion. Of the 23 patients who had a post-operative diagnosis of GBC following pathological examination of the resected specimens, 21 underwent simple cholecystectomy alone without any additional resections and 2 underwent radical resection additionally following simple cholecystectomy (open in 1 and laparoscopic in 1).

As a result, of the 47 patients, 29 (62%) underwent simple cholecystectomy (open 21 and laparoscopic 8), and 18 (38%) underwent radical resection with regional lymphadenectomy in this series (Table 1). In the 18 patients who underwent radical resection, 8 underwent full-thickness cholecystectomy (cholecystectomy combined with the removal of the entire connective tissue lying between the gallbladder and the liver parenchyma) with regional lymphadenectomy, 9 underwent extended cholecystectomy along with regional lymphadenectomy, including 8 who underwent wedge resection of the gallbladder bed and 1 who underwent wedge resection of the gallbladder bed in addition to bile duct resection. The remaining 1 patient with remnant cystic duct cancer (2.4 years following cholecystectomy for benign gallbladder disease) underwent bile duct resection and regional lymphadenectomy. Our department mainly applies full-thickness cholecystectomy to patients with advanced age and/or comorbid disease(s) when the tumour is apparently confined to the gallbladder wall. Regional lymph nodes of the gallbladder included the cystic duct, pericholedochal, posterior-superior pancreaticoduodenal, retroportal, right celiac, and hepatic artery node groups [13–16]. None of the patients received para-aortic lymph node dissection. There was no mortality until patients were discharged from the hospital following the surgical procedure. None of the patients experienced adjuvant chemotherapy or radiotherapy. Subsequently, the patients were consistently followed up in outpatient clinics every 3–6 months for at least 5 years, with imaging such as computed tomography on a regularly basis. The follow-up periods ranged from 6 to 440 (median, 144) months after surgical resection.

**Table 1:** Surgical procedures for 47 patients with T1b gallbladder cancer

Procedure	No. of patients
Simple cholecystectomy	
Open	21
Laparoscopic	8
Radical resection	
C^a^ + N	8
C + WR + N	8
C + WR + BD + N	1
C^a^ + BD + N	1

*C* Cholecystectomy, *N* Radical regional lymphadenectomy, *WR* Wedge resection of the gallbladder bed, *BD* Resection of the extrahepatic bile duct

^a^ Cholecystectomy with full-thickness dissection (cholecystectomy combined with the removal of the entire connective tissue lying between the gallbladder and the liver parenchyma)

### Histopathological evaluation

**Fig. 1:**
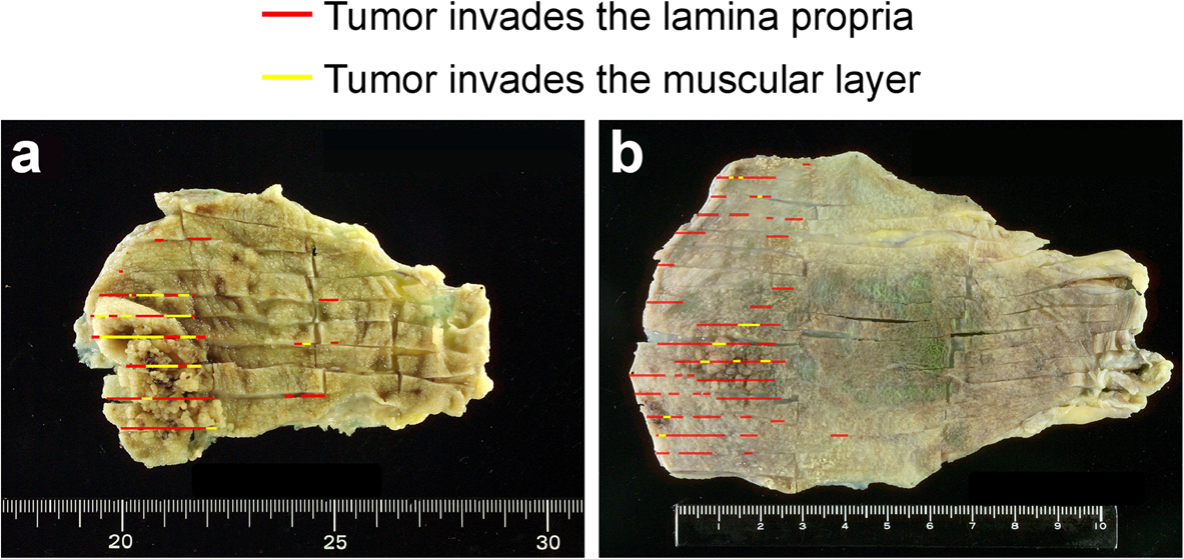
Precise pathological examination and macroscopic appearance of early-stage gallbladder cancer. The resected gallbladder specimen was sliced at 5 mm intervals, and precise pathological examinations were performed by mapping cancer lesions to reduce the possibility of pathological under-staging. **a** Protruding type, sessile tumour plus superficial elevated type. **b** Superficial elevated plus flat type. Red and yellow lines indicate the area of the tumour invading the lamina propria and muscular layer, respectively

The gallbladder specimens were sent to our pathology department for histopathological evaluation. Since 1982, a standard protocol has been implicated at our institution for the final histopathological description of gallbladder tumours for over 37 years. The degree of invasion was determined by studying numerous sections (range, 10 to 77 sections; median, 18 sections) of the resected specimen in individual cases. Once the diagnosis of early-stage GBC was established, the specimens were sliced at intervals of 5 mm, and precise pathological assessments were carried out via mapping of cancer lesions to scale back the potential for pathological under-staging (Fig. 1). The macroscopic appearance of early-stage GBC, including T1a and T1b tumours, was classified as protruding or superficial (Fig. 1) [17]. Histopathological findings were referred to based upon the American Joint Committee on Cancer (AJCC) Cancer Staging Manual [16]. The primary tumour was adenocarcinoma in 45 patients, adenosquamous carcinoma in 1 patient, and adenoneuroendocrine carcinoma in 1 patient. Histological grade was well differentiated in 34 patients, moderately differentiated in 8 patients and poorly differentiated in 5 patients. A total of 170 regional lymph nodes retrieved from 25 patients were inspected histologically to rule out metastasis. There was no evidence in any patient regarding macroscopic or microscopic enduring tumour margins.

### Prognostic factors

To elucidate variables impacting long-term results relating to resections, 7 variables were verified in all 47 patients: age (≤ 70 years vs. > 70 years), gender, surgical procedure (simple cholecystectomy vs. radical resection), surgical approach (open vs. laparoscopic), size of primary tumour (≤ 60 mm vs. > 60 mm), macroscopic type (protruding vs. superficial), and histological grade (G1 vs. G2 + G3).

### Statistical analysis

Medical data and survival details were obtained for all patients. Specific parameters were equated through Fisher’s exact test. The cause of death was identified from our databased medical archives. Deaths from other causes had been addressed as uncensored instances in the setting of overall survival analysis, while deaths from other factors were treated as censored cases in the setting of disease-specific survival analysis. The Kaplan-Meier method was utilized to calculate the collective incidences of events, and variations in these events were assessed with the log-rank test. A Cox proportional hazards regression model was carried out to recognize components that were individually related to overall survival and disease-specific survival. Within this model, a stepwise selection was applied for variable selection with entry and elimination limitations of *P* < 0.1 and *P* >  0.15, respectively. The steadiness of each model was verified with a step-backward and step-forward fitting procedure, and parameters identified as an impartial effect on overall survival and disease-specific survival were indistinguishable within the two procedures. All statistical evaluations were carried out by IBM SPSS Statistics 24 (IBM Japan, Inc., Tokyo, Japan). All tests were two-sided, and *P* values of < 0.05 were regarded as statistically significant.

## Results

### Extent of tumour spread in T1b GBC

There was no blood venous or perineural invasion upon histological examination of the patient specimens. The histological assessment of one patient’s specimen revealed lymphatic vessel invasion. Metastases were absent in all regional lymph nodes examined. The median number of lymph nodes examined histologically was 8 (range, 1 to 32) in 18 patients who underwent radical resection. None of the patients had clinically apparent distant metastasis during resection.

### Clinicopathological characteristics according to surgical procedure

The open surgical approach was more frequent in 18 patients who underwent radical resection (open in all 18 patients) than in the 29 patients who underwent simple cholecystectomy (open in 21; laparoscopic in 8) (*P* = 0.017). The other clinicopathological factors, including age, gender, size of primary tumour, macroscopic type, histological grade, lymphatic vessel invasion, blood venous invasion, perineural invasion, lymph node metastasis, and residual tumour status, were comparable between simple cholecystectomy and radical resection (Table 2).

**Table 2:** Clinicopathological characteristics of 47 patients with T1b gallbladder cancer according to surgical procedure

Variable	Simple cholecystectomy (*N* = 29)	Radical resection (*N* = 18)	*P* value
Age (≤ 70 years/> 70 years)	7/22	10/8	0.059
Gender (Male/Female)	13/16	3/15	0.062
Size of primary tumor (≤ 60 mm/> 60 mm)	20/9	10/8	0.371
Macroscopic type (Protruding/Superficial)	13/16	10/8	0.556
Surgical approach (Open/Laparoscopic)	21/8	18/0	0.017
Histological grade (G1/G2 + G3)	21/8	13/5	> 0.999
Lymphatic vessel invasion (Absent/Present)	29/0	17/1	0.383
Blood venous invasion (Absent/Present)	29/0	18/0	NA
Perineural invasion (Absent/Present)	29/0	18/0	NA
Lymph node metastasis (Absent/Present)	29/0	18/0	NA
Residual tumor status (R0/R1 + R2)	29/0	18/0	NA

*T1b* Tumor invades the muscular layer, *G1* Well differentiated, *G2* Moderately differentiated, *G3* Poorly differentiated, *R0* No residual tumor, *R1* Microscopic residual tumor, *R2* Macroscopic residual tumor, *NA* Not available

### Long-term outcomes after resection

During disease status assessment, 20 patients passed away naturally following resection without having any recurrence. Two patients had radical resection but expired due to tumour relapse 99 and 39 months following resection without local recurrence.

The original sites of recurrence for these two patients were the lungs as well as the liver remnant (distant through the gall bladder bed), which were detected 96 and 30 months following resection, respectively. Both primary tumours of the two patients did not show particularly aggressive biological characteristics in histological examination; no lymphatic vessel, blood vessel, and perineural invasion was found, histological type and grade were adenocarcinoma and well differentiated, respectively.

Another 20 patients (simple cholecystectomy in 13 patients and radical resection in 7 patients) passed away from other causes without any evidence of disease. Neither port site recurrence nor peritoneal seeding after laparoscopic cholecystectomy occurred in this series. The enduring 25 patients were alive devoid of any disease.

**Fig. 2:**
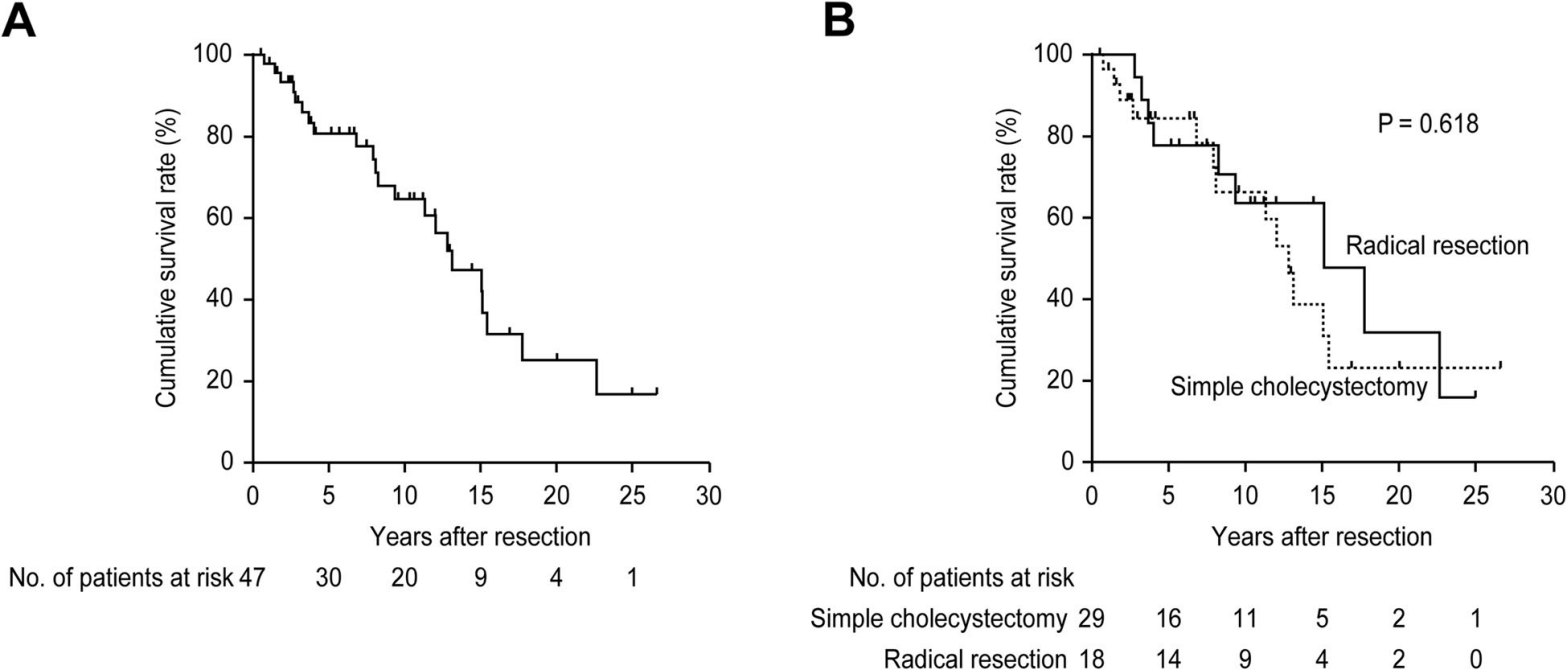
Kaplan-Meier estimates of overall survival in 47 patients with T1b gallbladder cancer. **a** The median overall survival time was 158 months with cumulative 5-, 10-, 15-, and 20-year overall survival rates of 81, 65, 48, and 25%, respectively. **b** The median overall survival time in patients undergoing simple cholecystectomy was 154 months with cumulative 5-, 10-, 15-, and 20-year survival rates of 84, 66, 40, and 24%, whereas the median overall survival time in patients undergoing radical resection was 182 months with cumulative 5-, 10-, 15-, and 20-survival rates of 78, 64, 64, and 32%, respectively (*P* = 0.618)

For all 47 patients, the cumulative 5-, 10-, 15-, and 20-year overall survival rates were 81, 65, 48, and 25%, respectively (Fig. 2 a); the median overall survival time was 158 months. The outcomes following simple cholecystectomy (10-year overall survival rate of 66%) were equivalent to those following radical resection (that of 64%, *P* = 0.618; Fig. 2 b). In this series, 8 patients undergoing a laparoscopic surgical approach had no evidence of bile spillage or perforation of the gallbladder during the operation. Among these 8 patients, 3 patients are alive devoid of disease, and 5 patients expired from other reasons unrelated to the disease. The outcome of the subsequent open surgical approach (10-year overall survival rate of 65%) was similar to that of the subsequent laparoscopic surgical approach (that of 64%, *P* = 0.139; Table 3).

**Table 3:** Univariate and multivariate analysis for overall survival in 47 patients undergoing surgical resection for T1b gallbladder cancer

Variable	Modality	No. of patients	Overall survival rate (%)		Univariate analysis *P* value	Multivariate analysis	
			5-year	10-year		Hazard ratio (95% CI)	*P* value
Age	≤ 70 years	17	94	94	0.005	1.000	
	> 70 years	30	72	45		5.285 (1.751–15.953)	0.003
Gender	Male	16	71	41	0.017	1.000	
	Female	31	86	76		0.272 (0.107–0.695)	0.007
Surgical procedure	Simple cholecystectomy	29	84	66	0.618		
	Radical resection	18	78	64			
Surgical approach	Open	39	80	65	0.139		
	Laparoscopic	8	86	64			
Size of primary tumor	≤ 60 mm	30	81	69	0.690		
	> 60 mm	17	81	59			
Macroscopic type	Protruding	23	74	60	0.743		
	Superficial	24	87	68			
Histological grade	G1	34	87	78	0.032		
	G2 + G3	13	65	33			

*T1b* Tumor invades the muscular layer, *CI* Confidence interval, *G1* Well differentiated, *G2* Moderately differentiated, *G3* Poorly differentiated

**Fig. 3:**
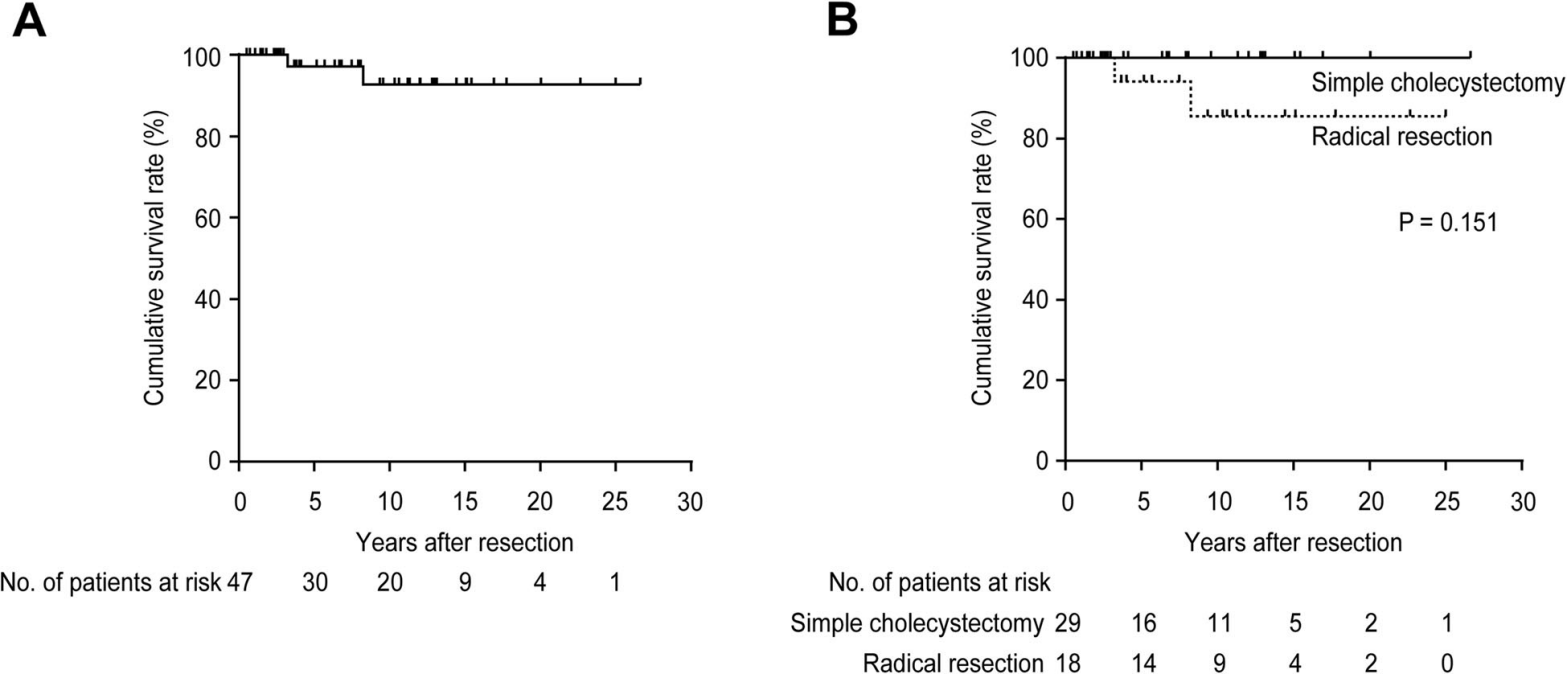
Kaplan-Meier estimates of disease-specific survival in 47 patients with T1b gallbladder cancer. **a** The median disease-specific survival time was not reached with cumulative 5-, 10-, 15-, and 20-year disease-specific survival rates of 97, 93, 93, and 93%, respectively. **b** The median disease-specific survival time in patients undergoing simple cholecystectomy was not reached with cumulative 5-, 10-, 15-, and 20-year disease-specific survival rates of 100, 100, 100, and 100%, whereas the median disease-specific survival time in patients undergoing radical resection was not reached with cumulative 5-, 10-, 15-, and 20-year disease-specific survival rates of 94, 86, 86, and 86%, respectively (*P* = 0.151)

For all 47 patients, the cumulative 5-, 10-, 15-, and 20-year disease-specific survival rates were 97, 93, 93, and 93%, respectively (Fig. 3 a). The results after simple cholecystectomy (10-year disease-specific survival rate of 100%) were not significantly different from those after radical resection (that of 86%, *P* = 0.151; Fig. 3 b). The outcome after the open surgical approach (10-year disease-specific survival rate of 92%) was comparable to that after the laparoscopic surgical approach (that of 100%; *P* = 0.592).

#### Prognostic factors after resection

The univariate analyses revealed that age (*P* = 0.005), gender (*P* = 0.017), and histological grade (*P* = 0.032) were statistically significant prognostic factors for overall survival (Table 3). Variables that were significant in the univariate analyses were entered into the multivariate analyses, which revealed that age (> 70 years, hazard ratio 5.285, *P* = 0.003) and gender (female, hazard ratio 0.272, *P* = 0.007) were independent prognostic factors for overall survival (Table 3). Surgical procedure (simple cholecystectomy vs. radical resection) and surgical approach (open vs. laparoscopic) did not affect inclusive survival in patients with T1b GBC.

## Discussion

To date, there are no accepted, robust treatment guidelines for T1b GBC. The NCCN guidelines endorse radical resection along with portal lymph node dissection for T1b GBC [9], whereas the Japanese guidelines recommend simple cholecystectomy, provided that the depth of invasion is histologically restricted to the muscular layer [10]. Thus, no consensus regarding the performance of radical resection for T1b GBC has been established. This fact motivated us to carry out the present research, which obviously indicated that age (> 70 years) and gender (male) had a robust undesirable impact on overall survival without any survival advantage of radical resection in patients with T1b GBC.

Controversy exists as to whether T1b GBC may have spread locally, regionally, or systemically by the timing of presentation. The frequency of lymph node metastasis in T1b GBC has been reported to range from 0 to 15.6% [4, 5, 8, 11, 18]. This difference in incidence may be due to histopathological examination, in which T-stage migration occurs, implying that some T2 GBCs may be diagnosed as T1b. In our previous study of 25 patients with T1b GBC on precise pathological examination (5 mm intervals of multiple tissue sections and mapping of cancer lesions to reduce the possibility of pathological understating), local spread of T1b GBCs was observed [11]. The current study is consistent with our previous results; most T1b GBCs are local disease provided that precise histopathological examination is performed.

The incidence of T1b GBC is comparatively minimal, which makes it challenging to gather an adequate number of cases to provide a treatment guideline for every single stage. The detection of early-stage GBC remains difficult [9]. In our retrospective morphological study of 299 resected specimens of early-stage GBCs, two-thirds of early-stage GBCs were classified as superficial, whereas one-third was classified as protruding [17]. Jang et al. [19] investigated the depth of invasion of T1 GBC, more than 1-cm polypoid GB lesions, pre-operatively by 2 modalities: high resolution ultrasonography (HRUS) and endoscopic ultrasonography (EUS), and they reported accuracy of prediction on HRUS was 69.2% and on EUS was 53.8%. These findings support the credence that it is difficult to distinguish T1a and T1b, especially superficial type, as the normal gallbladder wall is too thin for satisfactory evaluation, and the Rokitansky-Aschoff sinus adds to diagnostic difficulties. Nevertheless, HRUS provides excellent information because of its proficiency in distinguishing layered gallbladder wall anatomy [19]. In fact, in this series, pre-operative ultrasonographic judgement of the depth of invasion was not conceivable in any of the 16 patients who had pre-operative diagnosis of GBC by ultrasonography, and 38% of patients with T1b GBC received radical resection regardless of the absence or presence of lymph node metastasis. As pre-operative diagnosis including tumour penetration of T1b GBC remains difficult, the performance of radical resection is justified as a first definitive surgical procedure in select patients.

According to the NCCN guidelines, radical resection with portal lymph node dissection is recommended in patients with GBC above T1b [9]. According to the Japanese guidelines, resection is achievable simple cholecystectomy [10]. In this study, both overall survival (Fig. 2 b) and disease-specific survival (Fig. 3 b) were comparable between simple cholecystectomy and radical resection. In the multivariate analysis (Table 3), age (> 70 years) and gender (male) were found to have robust adversative effects on patient overall survival; on the other hand, no survival benefit of surgical procedure (simple cholecystectomy vs. radical resection) was shown in patients with T1b GBC. In the latest multicentral study by Kim et al. [5], incorporating 272 patients with T1b GBC revealed that simple cholecystectomy is acceptable, and there were no overall survival benefits or prevention of recurrence with extended or radical resection. Another systemic review by Lee et al. [8] advocated that a simple cholecystectomy including a laparoscopic approach is justifiable for T1b GBC. Our study results are consistent with the above-mentioned outcomes. In this study, once the diagnosis of early-stage GBC was established, the gallbladder specimens were sliced at 5 mm intervals, and precise pathological investigations were completed through mapping of cancer lesions to decrease the likelihood of pathological understating (Fig. 1). In regards to precise pathological examination, provided that the depth of invasion is restricted to the muscular layer and the surgical margins are uninvolved, the performance of simple cholecystectomy alone is justified as an adequate surgical procedure for T1b GBC.

Currently, it remains controversial whether open or laparoscopic surgical procedures have significant effects on oncological results. In the Japanese guidelines [10], an open approach is recommended for patients with pre-operatively diagnosed or suspected GBC because laparoscopic cholecystectomy has risks of port site relapse as well as peritoneal propagation [19, 20]. In the univariate analyses over long-term follow-up, the surgical approach was not associated with overall survival or disease-specific survival; in the multivariate analysis, no survival benefit of the surgical approach (open vs. laparoscopic) was recognized in patients with T1b GBC. Regardless of the surgical approach, disease-specific survival was excellent, with a cumulative 5-year disease-specific survival rate of 97% (92% in the open approach group and 100% in the laparoscopic approach group). Neither port site recurrence nor peritoneal seeding after laparoscopic cholecystectomy occurred in this series. The most recent study by Kim et al. [5], including 14 tertiary centres, stated that there was no difference in 5-year disease-specific survival among patients with open cholecystectomy and laparoscopic cholecystectomy (94.9% vs. 92.8%, respectively, *P* = 0.267), without any trocar-site recurrence in the laparoscopic cholecystectomy group. Professional surgeons with a great deal of expertise in laparoscopic cholecystectomy and careful handling of specimens using retrieval bags will encourage the safety of laparoscopic procedures in GBCs [5]. However, it would be interesting to gauge the impact of incidental iatrogenic perforation of the gallbladder in relation to long-term outcomes of patients with T1b GBC. The learning curve of sophisticated instruments coupled with expertise in laparoscopy remains the key factors to avoid perforation and subsequently reduce the risk of port site recurrence. Proper handling of specimens with retrieval bags and expertise in laparoscopy can offset this issue. In the present study, there is a paucity of data regarding the laparoscopic approach for T1b GBC. Taken together, these results suggest that surgical outcome is excellent for T1b GBC regardless of surgical approach, while the issue regarding surgical approach (open vs. laparoscopic) is still under debate for GBC above T1b.

The cystic duct has been included in the staging classification scheme of GBC since the 7th edition of the AJCC Cancer Staging Manual was published in 2010 [21]. In this study, we encountered a patient with remnant cystic duct cancer (2.4 years after simple cholecystectomy for benign gallbladder disease); the patient underwent bile duct resection and regional lymphadenectomy and passed away after 4 years following the second operation. For T1a GBC, Shirai et al. [4] found that 2 patients with residual carcinoma in situ at the cystic ductal stumps expired of local recurrence at 66 months and 76 months, respectively, later enduring cholecystectomy. Thus, resection of the extrahepatic bile duct is warranted for cystic duct origin GBC or GBC involving the cystic duct in order to attain a negative surgical margin (no residual tumour). Leading benefit of positron emission tomography-computed tomography (PET- CT) over multi-detector row CT (MDCT) is its capability to identify residual tumor and/or occult metastatic disease in the remainder of the entire body instead of MDCT of the abdomen and pelvis which can be beneficial in the localized, regional and/or distant staging of the disease [22]. Inclusion of PET-CT to standard cross-sectional imaging incorporates a moderate influence on disease management pre-operatively, specifically in patients with no preceding cholecystectomy and also to verify dubious nodal disease on CT [23].

There are several limitations in this study. First, the primary constraints of this research were its retrospective analysis using a small group of patients. Second, the follow-up time period in 9 patients was less than 60 months. Therefore, we could not reach definitive conclusions as to whether T1b GBC may spread loco-regionally and whether radical resection is necessary. However, to our knowledge, the existing research is probably the most significant series of patients with T1b GBC with a long-term follow-up period that describes outcomes of the surgical procedure and approach and its impact on overall survival. Also, one of the strengths of this study is that histological examination of surgical specimens was consistently uniform throughout the study period. These indicate more clearly than previous studies regarding the tumour characteristics and treatment of T1b GBC.

## Conclusions

In conclusion, most T1b GBCs spread only locally. As pre-operative diagnosis, including tumour penetration of T1b GBC, is difficult, the decision of radical resection is justified. Our study with the small number of patients over a large time period revealed that added radical resection may not be essential after simple cholecystectomy provided that the depth of invasion is restricted to the muscular layer and that surgical margins are uninvolved.

## Data Availability

The datasets utilized and/or reviewed throughout the present study can be obtained from the corresponding author on sensible request.

## Abbreviations

AJCC: American joint committee on cancer
EUS: Endoscopic ultrasonography
GBC: Gallbladder cancer
HRUS: High resolution ultrasonography
NCCN: National comprehensive cancer network
